# Whole body balance control in Lenke 1 thoracic adolescent idiopathic scoliosis during level walking

**DOI:** 10.1371/journal.pone.0229775

**Published:** 2020-03-06

**Authors:** Kuan-Wen Wu, Tung-Wu Lu, Wei-Chun Lee, Ya-Ting Ho, Jyh-Horng Wang, Ken N. Kuo, Ting-Ming Wang

**Affiliations:** 1 Department of Biomedical Engineering, National Taiwan University, Taipei, Taiwan, R.O.C; 2 Department of Orthopaedic Surgery, School of Medicine, National Taiwan University, Taipei, Taiwan, R.O.C; 3 Department of Orthopaedic Surgery, National Taiwan University Hospital, Taipei, Taiwan, R.O.C; 4 Department of Orthopaedic Surgery, Chang Gung Memorial Hospital, Taipei, Taiwan, R.O.C; Texas State University, UNITED STATES

## Abstract

**Introduction:**

Altered trunk shape and body alignment in Lenke 1 thoracic adolescent idiopathic scoliosis (AIS) may affect the body’s balance control during activities. The current study aimed to identify the effects of Lenke 1 thoracic AIS on the balance control during level walking in terms of the inclination angles (IA) of the center of mass (COM) relative to the center of pressure (COP), the rate of change of IA (RCIA), and the jerk index of IA. The association between the Cobb angle, IA and RCIA was also evaluated.

**Materials and methods:**

Sixteen adolescents with AIS (age: 14.0±1.8 years, height: 154.8±4.7 cm, mass: 42.0±7.5 kg) and sixteen healthy controls (age: 14.4±2.0 years, height: 158.4±6.2 cm, body mass: 48.6±8.9 kg) performed level walking in a gait laboratory. The kinematic and ground reaction force data were measured for both concave-side and convex-side limb cycles, and used to calculate the IA and RCIA, the jerk index of IA, and the temporal-spatial parameters. Correlations between the Cobb angle, IA and RCIA were quantified using Pearson’s correlation coefficients (r).

**Results:**

The patients showed less smooth COM-COP motion with increased jerk index of IA in the sagittal plane during single limb support (SLS) of the concave-limb (p = 0.05) and in the frontal plane during double limb support (DLS) (p < 0.05). The patients also showed significantly increased posterior RCIA on both the concave and convex side during initial (p = 0.04, p = 0.03) and terminal (p = 0.04, p = 0.03) DLS when compared to healthy controls. In the frontal plane, the patients walking on the concave-side limb showed decreased IA over SLS (p = 0.01), and at contralateral toe-off (p<0.01) and contralateral heel-strike (p = 0.02), but increased mean IA magnitude over terminal DLS (p = 0.01). The frontal IA at contralateral toe-off and SLS for AIS-A showed a moderate to strong correlation with Cobb angles (r = -0.46 and -0.61), and the sagittal RCIA over the initial DLS for AIS-A also showed a significant, strong correlation with Cobb angles (r = -0.50).

**Conclusions:**

The patients with Lenke 1 thoracic scoliosis in the current study showed altered and jerkier COM-COP control during level walking when compared to healthy controls. During DLS, the patients increased the posterior RCIA in the sagittal plane with increased IA jerk index in the frontal plane for both the concave- and the convex-side limb, indicating their difficulty in maintaining a smooth transfer of the body weight. During SLS of the concave-side limb, the patients adopted a conservative COM-COP control strategy, as indicated by a decreased IA in the frontal plane, but showed a jerky COM-COP control in the sagittal plane. The COM-COP control of the patients was associated with the severity of the spinal deformity. The current results suggest that this patient group should be monitored for signs of an increased risk of loss of balance during weight transfer on the concave-side limb.

## Introduction

Adolescent idiopathic scoliosis (AIS) is a three-dimensional spinal deformity during adolescence with an overall prevalence of 0.47–0.52% [1]. Scoliosis is characterized by a lateral spinal curvature with an axial rotation [2, 3], and is classified into six curve types depending on the regions involved (proximal thoracic, main thoracic, or thoracolumbar/lumbar) [4]. Among these types, Lenke 1 involves a single main lateral curve of T5-T12, and has the highest incidence [4–8]. The deformed thoracic spinal curve has been shown to lead to compromised postural stability with an increased amount of static sway area during standing [9, 10], as well as poor body segmental alignment during dynamic activities associated with increased center of mass (COM) displacement [11], and altered motions of the trunk and other segments [12–17].

Kinematic changes of the body segments associated with AIS are thought to be a compensatory mechanism for maintaining whole-body dynamic balance [18–20]. Asymmetrical AIS spinal deformity alters the shape, mass distribution and thus the COM position of the trunk [21], the extent of which depends on the severity of the condition. With the upper body accounting for about 60% of the body’s mass, the altered trunk inertial properties may further affect the motions of the trunk itself and other body segments in order to maintain balance during activities such as gait [14, 22], which are expected to increase the neuromechanical demand on the locomotor system. Such compensatory changes in the control of the motions of the body segments may be different between gait cycles of the convex and concave sides. On the other hand, since AIS often occurs with impaired proprioception [23], maintaining balance during level walking can be more difficult than for healthy peers. Therefore, monitoring balance control during activities may provide useful information for the clinical management of this patient population.

The body’s balance control can be quantified by the motion of the body’s COM relative to the center of pressure (COP) in terms of the COM-COP inclination angles (IA), the angles formed by the vertical line and the line connecting the COP and COM, and the rate of change of IA (RCIA) [24, 25]. During walking, the COM can be outside the constantly changing and moving base of support (BOS) and far away from the COP without loss of balance as long as the COM is controlled at an appropriate velocity relative to the COP [26]. Generally, the further the COM diverges from the COP (i.e., greater IA), the more difficult it becomes, and more effort (e.g., joint torque) is needed to achieve an RCIA appropriate for dynamic balance [26]. In other words, an increased IA may not suggest poor balance control as long as one is able to generate an appropriate RCIA either by changing the velocity of the COM or that of the COP, or both. Therefore, interpretation of the IA data of a patient group should be made together with RCIA and with reference to those of healthy controls for a more complete assessment of the balance control. The IA and RCIA together have been used in the study of balance control strategies in various populations during dynamic activities [27–29]. However, there is only one study on the balance control of patients with AIS in terms of COM-COP motions pre- and post-spinal fusion surgery [26]. The results focused more on the COM displacements and did not include age-matched healthy controls, limiting the correct interpretation of the results. To the best knowledge of the authors, no study has investigated the effects of severe Lenke 1 AIS on the balance control and its association with the severity of the deformity in terms of Cobb angle, COM-COP IA and RCIA during level walking when compared to healthy controls, and comparing between sides.

The purpose of this study was to identify the effects of thoracic spinal deformity on whole-body balance control during level walking, between-side differences and their association with the severity of the spinal deformity, in terms of COM-COP inclination angles in patients with Lenke 1 thoracic AIS. It was hypothesized that patients with Lenke 1 thoracic AIS would walk with altered COM-COP control with a more jerky transfer of the body weight when compared to healthy controls, and that more conservative balance control, as indicated by a reduced IA would be adopted during the concave-side limb cycle than during the convex-side limb cycle, and that the level of balance control was associated with the severity of the spinal deformity.

## Methods

Sixteen female adolescents with thoracic AIS (AIS group; age: 14.0±1.8 years, height: 154.8±4.7 cm, mass: 42.0±7.5 kg) participated in the current study with informed written consent signed by the subjects and their legal guardians as approved by the Institutional Research Board. All the patients were determined radiographically to have a Lenke 1 thoracic curve [30] with Cobb angles of 54.6±14.5° and kyphosis angles of 28.2±9.1°. They were of normal lower-limb muscle strength, as assessed by Manual Muscle Tests [31], with corrected vision and right-leg dominance, without physical limitations in performing daily or sports activities. The only treatment received was wearing a brace during the daytime. All the assessments and experimental measurements in the current study were performed without the brace. Participants were excluded if they had a neurological disorder, leg length discrepancies greater than 1 cm or other musculoskeletal diseases, such as trauma, muscle atrophy or joint diseases that would affect their gait performance. Sixteen healthy adolescents (Control group; age: 14.4±2.0 years, height: 158.4±6.2 cm, body mass: 48.6±8.9 kg) were selected to match with the AIS group for sex, age and BMI. An *a priori* power analysis based on pilot results of sagittal and frontal IA and RCIA from three patients with AIS and three healthy subjects using GPOWER 3 [32] determined that a projected sample size of eleven subjects for each group would be enough for a power of 0.8 and an effect size of 1.7 at a significance level of 0.05. Thus, sixteen subjects for each, namely the AIS and the control group, were adequate for achieving the main objectives of this observational, cross-sectional study.

In a gait laboratory, each subject walked at a self-selected pace on a 10-meter walkway while thirty-nine infrared retro-reflective markers were used to track the motions of the body segments. These markers were placed on the anterior superior iliac spines (ASISs), posterior superior iliac spines (PSISs), greater trochanters, mid-thighs, medial and lateral epicondyles, heads of fibulae, tibial tuberosities, medial and lateral malleoli, navicular tuberosities, fifth metatarsal bases, big toes and heels, and mandibular condylar processes, acromion processes, C7, medial and lateral humeral epicondyles, and ulnar styloids [14, 33, 34]. Three-dimensional trajectories of the markers were measured using an 8-camera motion analysis system (Vicon MX T-40, OMG, U.K.) at 120 Hz, and the ground reaction forces were measured using three forceplates (464 mm x 508 mm, OR6-7, AMTI, U.S.A.) at 1080 Hz. Data from three complete gait cycles for each lower limb from six trials were obtained for each subject. For the AIS group, the gait cycles of the convex-side limb were denoted AIS-V while those of the concave-side limb were denoted AIS-A.

The body’s COM position was calculated as the weighted sum of the positions of the COMs of all the body segments using the marker data and segmental inertial properties. Effects of soft tissue artifacts of the pelvis-leg apparatus were reduced using a global optimization method that minimized the weighted sum of squared distances between measured and calculated marker positions [35]. Subject-specific body segmental inertial properties were obtained using an optimization-based method, which has been shown to reduce errors in the calculated center of mass motions and joint moments when compared to commonly-used prediction methods [36]. The COP position was calculated using forces and moments measured by the forceplates. The gait events of heel-strike and toe-off were determined from the forceplate data [37, 38]. Spatiotemporal parameters of gait, namely stride length, stride time, step length, step width, cadence and gait speed were also obtained. The COM-COP inclination angles (IA) in the sagittal and frontal planes were then calculated as follows.

$$\overset{⃑}{t}=\left(\overset{⃑}{Z}\times \frac{{\overset{⃑}{P}}_{COM-COP}}{\left|{\overset{⃑}{P}}_{COM-COP}\right|}\right)$$ (1)

$$\mathrm{Sagittal}\;\mathrm{IA}=\sin^{-1}\left(t_{Y}\right)$$ (2)

$$\mathrm{Frontal}\;\mathrm{IA}=\left\{ \begin{array}{c} {-\sin}^{-1}(t_{X}),\;for\;the\;right\;limb\\ \sin^{-1}\left(t_{X}\right),\;for\;the\;left\;limb \end{array}\right.$$ (3)

where ${\overset{⃑}{P}}_{COM-COP}$ was the vector pointing from the COP to the COM, $\overset{⃑}{Z}$ was the unit vector of the vertical and $\overset{⃑}{X}$ was the unit vector pointing in the direction of progression. With the current forceplate setup, the IAs were calculated from the beginning of swing phase of the leading limb to the subsequent contralateral heel-strike. The RCIA was calculated by smoothing and differentiating the IA trajectories using the GCVSPL (Generalized Cross-Validatory SPLine) package [39]. For the leading limb, positive sagittal and frontal IA indicate that the COM is anterior to and away from the COP toward the contralateral limb, respectively. The smoothness of the COM-COP control was quantified by the jerk index, which was calculated for bilateral single-limb support (SLS) and double-limb support (DLS) using the third derivatives of the IA trajectories as follows [40–42].

$$Jerk\;Index=\int_{t_{i}}^{t_{f}}{\left({IA}^{\prime \prime \prime }\right)}^{2}\;dt$$ (4)

where *t_i_* and *t_f_* are, respectively, the beginning and end of the sub-phase considered.

For statistical analysis, the values of the IA and RCIA at heel-strike, toe-off, and contralateral heel-strike and toe-off were obtained for each of the AIS-V and AIS-A gait cycles for the AIS group, while those for the Control group were obtained from gait cycles of both sides. The range of motion of IA during the gait cycle, and time-averaged IA over DLS and SLS, as well as the peak RCIA during DLS, and time-averaged RCIA over DLS and SLS were also obtained. For each calculated variable, data from three trials were averaged for each of AIS-V and AIS-A, while those from both sides (i.e., six trials) were averaged for Control. All the calculated variables were checked for normality of distribution using a Shapiro-Wilk test. For variables of normal distribution, independent *t*-tests were used to compare the differences between AIS-V and Control and between AIS-A and Control, while paired *t*-tests were used to compare between AIS-V and AIS-A. For those variables with a non-normal distribution, Wilcoxon rank sum tests were used for between-group comparisons, while Wilcoxon signed rank tests were used to detect the differences between AIS-V and AIS-A. The association between the Cobb angle, IA and RCIA were evaluated using Pearson’s correlation coefficient (r). Absolute values of r less than 0.2 indicate a very weak correlation, 0.2–0.39 as weak, 0.40–0.59 as moderate, 0.6–0.79 as strong and 0.8–1 as very strong. A significance level of α = 0.05 was set for all tests. All statistical analyses were performed using SPSS version 20 (SPSS Inc., Chicago, IL, U.S.A.).

## Results

No significant differences were found in the walking speed between AIS and Control (Table 1). Compared to the Control, both AIS-A and AIS-V showed significantly increased single-limb support time, but the initial DLS time in AIS-V (i.e., terminal DLS time in AIS-A) was significantly decreased (Table 1).

**Table 1:** Means (standard deviations) of the spatiotemporal parameters during walking in the adolescent idiopathic scoliosis group (AIS, n = 16) and healthy controls (Control, n = 16).

Variables	AIS-V	AIS-A	Control	(P_V_, P_A_, P_S_)
Walking speed (mm/s)	1101.8 (166.1)	1126.8 (134.6)	1087.9 (82.0)	(0.77, 0.33, 0.47)
Stride length (mm)	1110.2 (76.1)	1115.1 (59.6)	1114.1 (46.2)	(0.86, 0.96, 0.69)
Step width (mm)	94.8 (38.2)	101.5 (32.4)	94.5 (28.8)	(0.98, 0.52, 0.25)
Cadence (steps/min)	117.4 (10.6)	118.1 (12.4)	113.9 (5.6)	(0.25, 0.23, 0.80)
Step length (mm)	587.4 (55.2)	583.2 (37.4)	585.5 (31.5)	(0.91, 0.85, 0.57)
Single limb support time (%)	39.3 (1.8)	39.7 (2.0)	37.2 (0.8)	(0.00*, 0.00*,0.18)
Initial double limb support time (%)	11.5 (1.6)	12.1 (2.0)	12.8 (0.7)	(0.01*, 0.19, 0.09)
Terminal double limb support time (%)	12.1 (2.0)	11.5 (1.6)	12.8(0.7)	(0.19, 0.01*, 0.09)
Contralateral single limb support time (%)	39.7 (2.0)	39.6 (1.8)	37.2 (0.8)	(0.00*, 0.00*,0.82)

AIS-V: cycle of the convex-side limb; AIS-A: cycle of the concave-side limb; P_V_ = AIS-V vs. Control; P_A_ = AIS-A vs. Control; P_S_ = AIS-A vs. AIS-V.

*: significant group effect; †: significant difference between sides (P_S_ < 0.05); HS: heel-strike; CTO: toe-off of contralateral limb; CHS: heel-strike of the contralateral limb; TO: toe-off.

**Fig 1:**
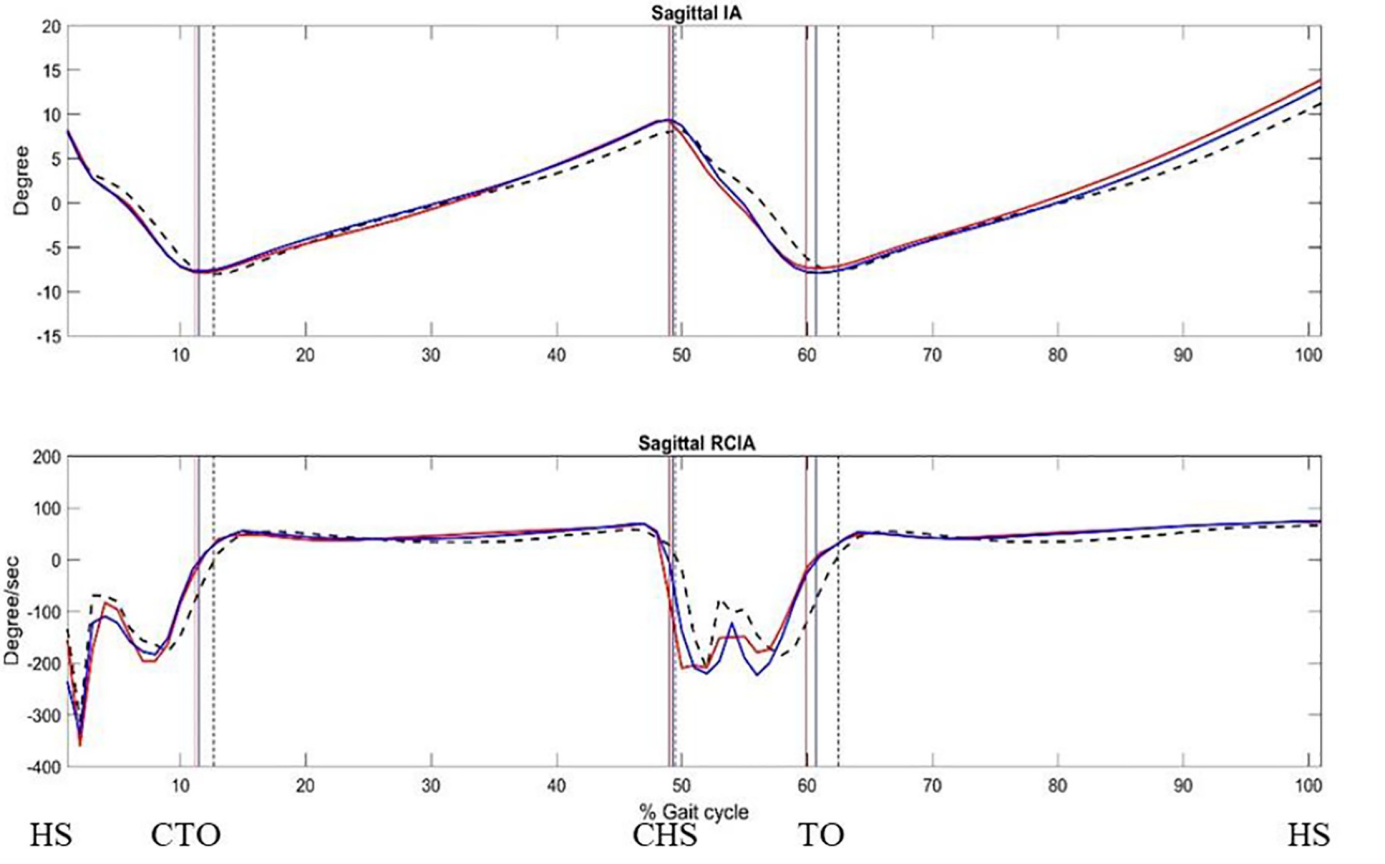
Ensemble-averaged sagittal COM-COP inclination angle (IA), and sagittal rate of change of IA (RCIA) during level walking: convex-side limb leading (red line), concave-side limb leading (blue line) and controls (dashed). The medial/lateral (M/L) positions of the COM and COP were described relative to the line of progression that described the M/L range of motion of the COM during a gait cycle, a positive value being to the side of the contralateral limb. (HS: heel-strike; CTO: toe-off of contralateral leg; CHS: heel-strike of the contralateral leg; TO: toe-off.) Positive sagittal and frontal IAs indicate COM positions that are anterior and contralateral to the COP, respectively.

In the sagittal plane, no significant differences between AIS and Control were found for any of the IA-related variables, but both AIS-V and AIS-A showed significantly increased posterior RCIA during initial and terminal DLS (Fig 1 and Table 2). Compared to AIS-V, AIS-A showed decreased posterior IA at contralateral toe-off but increased posterior IA at ipsilateral toe-off. AIS-A also showed an increased IA jerk index over SLS when compared to Control (Table 3).

**Table 2:** Means (standard deviations) of the RCIA (°/s) during walking in the adolescent idiopathic scoliosis group (AIS) and healthy controls.

	Rate of change of COM-COP inclination angle (RCIA,°/s)			
	AIS-V	AIS-A	Control	(P_V_, P_A_, P_S_)
Sagittal Plane				
CTO	-31.2 (94.7)	-37.1 (89.1)	3.8 (39.0)	(0.19, 0.11, 0.69)
CHS	-91.8 (127.3)	-76.7 (95.2)	-63.6 (66.1)	(0.44, 0.66, 0.51)
TO	-37.1 (89.1)	-31.2 (94.7)	3.8 (38.9)	(0.11, 0.19, 0.69)
Average DLSi	-149.9 (42.6)	-148.6 (42.1)	-122.7 (22.2)	(0.03*, 0.04*, 0.82)
Average SLS	47.8 (6.4)	47.3 (6.1)	44.8 (5.3)	(0.15, 0.23, 0.28)
Average DLSt	-148.6 (42.1)	-149.9 (42.3)	-122.7 (22.2)	(0.04*, 0.03*, 0.82)
Average CSLS	47.3 (6.1)	47.8 (6.4)	44.8 (5.3)	(0.23, 0.15, 0.28)
Peaki	-366.1 (144.8)	-368.2 (126.5)	-319.8 (88.4)	(0.28, 0.22, 0.93)
Peakt	-368.2 (126.5)	-366.1 (144.8)	-319.8 (88.4)	(0.22, 0.28, 0.93)
Frontal Plane				
CTO	21.1 (25.2)	21.7 (24.8)	12.9 (11.3)	(0.25, 0.21, 0.88)
CHS	-37.6 (40.3)	-32.4 (22.9)	-32.8 (16.3)	(0.66, 0.96, 0.47)
TO	-21.7 (24.8)	-21.1 (25.2)	-12.9 (11.3)	(0.21, 0.25, 0.88)
Average iDLS	52.9 (13.3)	48.2 (15.4)	48.8 (6.9)	(0.29, 0.89, 0.19)
Average SLS	0.3 (1.6)	1.0 (1.3)	0.5 (1.5)	(0.68, 0.37, 0.06)
Average tDLS	-48.2 (15.4)	-52.9 (13.3)	-48.8 (6.9)	(0.89, 0.29, 0.19)
Average CSLS	-1.0 (1.3)	-0.3 (1.6)	-0.5 (1.5)	(0.37, 0.68, 0.06)
Peaki	112.4 (42.7)	103.4 (32.2)	106.0 (17.7)	(0.59, 0.78, 0.46)
Peakt	-103.4 (32.2)	-112.4 (42.7)	-106.0 (17.7)	(0.78, 0.59, 0.46)

AIS-V: cycle of the convex-side limb; AIS-A: cycle of the concave-side limb; P_V_ = AIS-V vs. Control; P_A_ = AIS-A vs. Control; P_S_ = AIS-A vs. AIS-V.

*: significant group effect; †: significant difference between sides (P_S_ < 0.05); HS: heel-strike; TO: toe-off; CHS: contralateral heel-strike; CTO: contralateral toe-off; iDLS: initial double-limb support; tDLS: terminal double-limb support; SLS: single-limb support; CSLS: contralateral SLS.

**Table 3:** Means (standard deviations, SD) of the jerk index (10^5^°/s^3^) of the sagittal and frontal IA in the patients with adolescent idiopathic scoliosis and healthy controls during walking.

	AIS-V		AIS-A		Control		(P_V_, P_A_, P_S_)
Sagittal Plane							
DLSi	34.8	(19.6)	36.6	(28.7)	28.5	(15.1)	(0.32,0.33,0.78)
SLS	3.5	(3.8)	4.1	(4.4)	1.7	(0.5)	(0.09,0.05*,0.20)
DLSt	36.6	(28.7)	34.8	(19.6)	28.5	(15.1)	(0.33,0.32,0.78)
CSLS	4.1	(4.4)	3.5	(3.8)	1.7	(0.5)	(0.05*,0.09,0.20)
Frontal Plane							
DLSi	14.2	(12.3)	14.1	(13.6)	5.2	(3.3)	(0.01*,0.02*,0.97)
SLS	0.9	(1.0)	1.1	(1.3)	0.5	(0.2)	(0.18,0.13,0.55)
DLSt	14.1	(13.6)	14.2	(12.3)	5.2	(3.3)	(0.02*,0.01*,0.97)
CSLS	1.1		(1.3)	0.9		(1.0)	0.5		(0.2)	(0.13,0.18,0.55)

AIS-V: cycle of the convex-side limb; AIS-A: cycle of the concave-side limb; P_V_ = AIS-V vs. Control; P_A_ = AIS-A vs. Control; P_S_ = AIS-A vs. AIS-V.

*: significant group effect; DLSi: initial double-limb support; DLSt: terminal double-limb support; SLS: single-limb support; CSLS: contralateral SLS.

**Fig 2:**
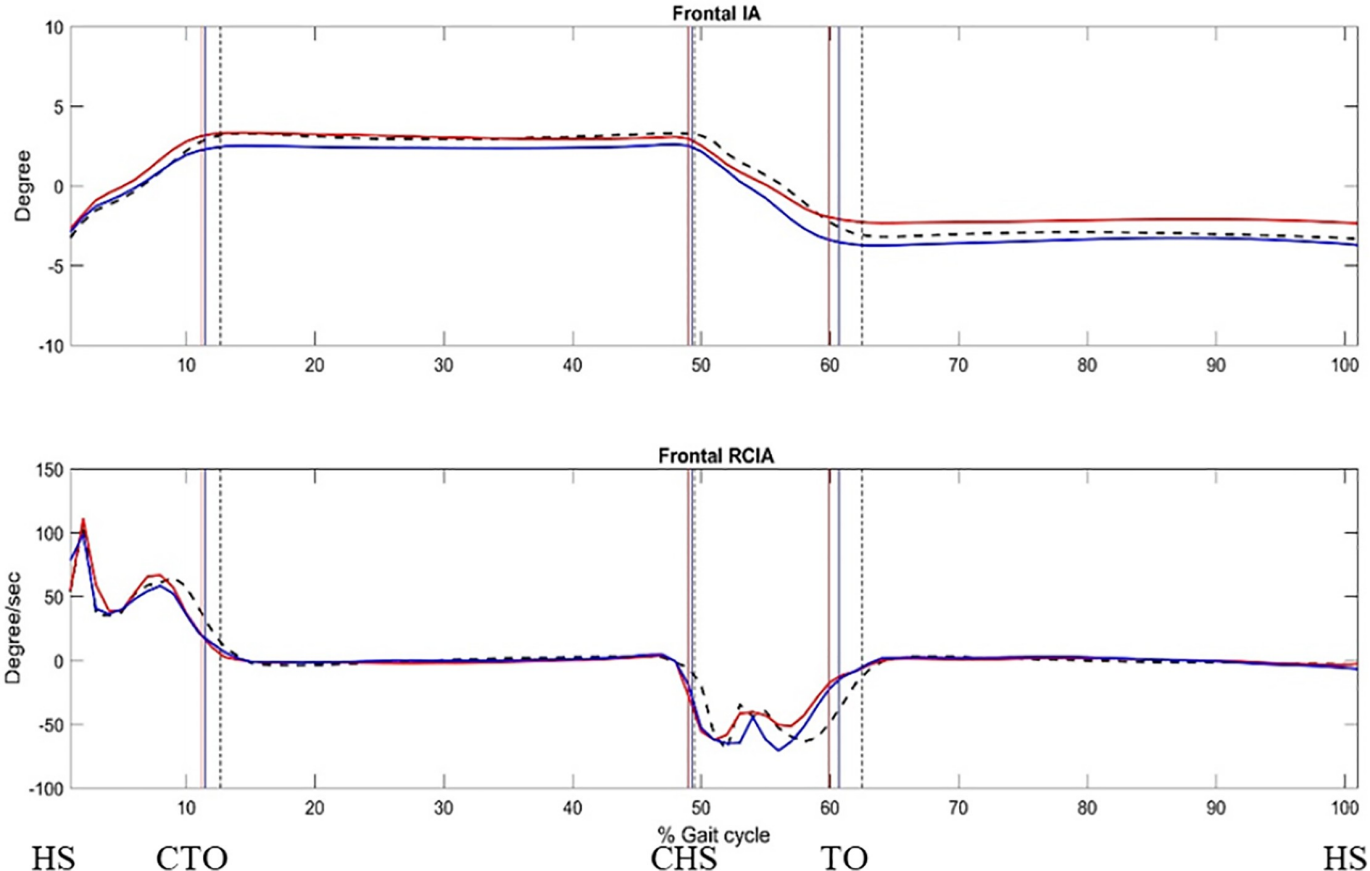
Ensemble-averaged frontal COM-COP inclination angle (IA), and frontal rate of change of IA (RCIA) during level walking: convex-side limb leading (red line), concave-side limb leading (blue line) and controls (dashed). The medial/lateral (M/L) positions of the COM and COP were described relative to the line of progression that described the M/L range of motion of the COM during a gait cycle, a positive value being to the side of the contralateral limb. (HS: heel-strike; CTO: toe-off of contralateral leg; CHS: heel-strike of the contralateral leg; TO: toe-off.) Positive sagittal and frontal IAs indicate COM positions that are anterior and contralateral to the COP, respectively.

In the frontal plane, compared to Control, the AIS-A showed significantly decreased IA over SLS and at contralateral (AIS-V) toe-off and heel-strike (Fig 2 and Table 4). Compared to AIS-V, the AIS-A showed significantly decreased IA over SLS and at contralateral toe-off and heel-strike, but significantly increased mean IA magnitude over terminal DLS, swing (contralateral SLS) and at ipsilateral toe-off (Table 4). Over initial and terminal DLS, both AIS-V and AIS-A showed an increased IA jerk index when compared to Control (Table 3).

**Table 4:** Means (standard deviations) of the IA (°) during walking in the adolescent idiopathic scoliosis group (AIS) and healthy controls.

	COM-COP inclination angle (IA,°)			
	AIS-V	AIS-A	Control	(P_V_, P_A_, P_S_)
Sagittal Plane				
CTO	-8.4 (1.4)	-7.9 (1.3)	-8.0 (1.1)	(0.42, 0.73, 0.03^†^)
CHS	8.4 (1.6)	8.7 (1.7)	8.2 (1.0)	(0.57, 0.30, 0.32)
TO	-7.9 (1.3)	-8.4 (1.1)	-8.0 (1.1)	(0.73, 0.42, 0.03^†^)
Average DLSi	-0.6 (1.5)	-0.7 (1.4)	-0.2 (0.8)	(0.34, 0.24, 0.77)
Average SLS	0.1 (1.1)	0.5 (1.5)	0.3 (0.4)	(0.51, 0.71, 0.11)
Average DLSt	-0.7 (1.4)	-0.6 (1.5)	-0.2 (0.8)	(0.24, 0.34, 0.77)
Average CSLS	0.5(1.5)	0.1 (1.1)	0.3 (0.4)	(0.71, 0.51,0.11)
Frontal Plane				
CTO	3.3 (0.9)	2.3 (0.8)	3.2 (0.5)	(0.47, 0.00*, 0.00^†^)
CHS	3.0 (0.7)	2.6 (0.9)	3.3 (0.6)	(0.34, 0.02*, 0.03^†^)
TO	-2.3 (0.8)	-3.3 (0.9)	-3.2 (0.5)	(0.00*, 0.47, 0.00^†^)
Average iDLS	0.4 (0.4)	-0.1 (0.4)	-0.0 (0.4)	(0.01*, 0.56, 0.00^†^)
Average SLS	3.3 (0.7)	2.4 (0.8)	3.1 (0.5)	(0.33, 0.01*, 0.00^†^)
Average tDLS	0.1 (0.4)	-0.4 (0.4)	0.0 (0.4)	(0.56, 0.01*, 0.00^†^)
Average CSLS	-2.4 (0.8)	-3.3 (0.7)	-3.1 (0.5)	(0.01*, 0.33, 0.00^†^)

AIS-V: cycle of the convex-side limb; AIS-A: cycle of the concave-side limb; P_V_ = AIS-V vs. Control; P_A_ = AIS-A vs. Control; P_S_ = AIS-A vs. AIS-V.

*: significant group effect

†: significant difference between sides (P_S_ < 0.05); HS: heel-strike; TO: toe-off; CHS: contralateral heel-strike; CTO: contralateral toe-off; iDLS: initial double-limb support; tDLS: terminal double-limb support; SLS: single-limb support; CSLS: contralateral SLS.

The frontal IA at toe-off for AIS-V (i.e., contralateral toe-off for AIS-A) was found to have a strong correlation with Cobb angles (r = -0.61, p = 0.01) (Table 5). In the sagittal plane, the RCIA over the initial DLS for AIS-A (i.e., terminal DLS for AIS-V) also showed a significant, strong correlation with Cobb angles (r = -0.50, p = 0.05) (Table 6). No significant correlations were found between Cobb angles and the other variables.

**Table 5:** Pearson's r of the IA (°) and Cobb angles during walking in the adolescent idiopathic scoliosis group (AIS).

COM-COP inclination angle (IA,°)				
	AIS-V	P	AIS-A	P
Sagittal Plane				
CTO	0.12	0.67	0.28	0.29
CHS	0.44	0.09	0.37	0.16
TO	0.28	0.29	0.12	0.67
Average DLSi	0.23	0.39	0.45	0.08
Average DLSt	0.45	0.08	0.23	0.39
Average SLS	0.24	0.36	0.26	0.34
Average CSLS	0.26	0.34	0.24	0.36
Frontal Plane				
CTO	0.14	0.60	-0.61	0.01*
CHS	-0.29	0.28	-0.030	0.26
TO	0.61	0.01*	-0.14	0.60
Average DLSi	0.13	0.62	-0.41	0.11
Average DLSt	0.41	0.11	-0.13	0.62
Average SLS	0.19	0.49	-0.46	0.07
Average CSLS	0.46	0.07	-0.19	0.49

AIS-V: cycle of the convex-side limb; AIS-A: cycle of the concave-side limb; P_V_ = AIS-V vs. Control; P_A_ = AIS-A vs. Control; P_S_ = AIS-A vs. AIS-V.

*: significant group effect; †: significant difference between sides (P_S_ < 0.05); HS: heel-strike; TO: toe-off; CHS: contralateral heel-strike; CTO: contralateral toe-off; iDLS: initial double-limb support; tDLS: terminal double-limb support; SLS: single-limb support; CSLS: contralateral SLS.

**Table 6:** The Pearson's r of the RCIA (°/s) and Cobb angles during walking in the adolescent idiopathic scoliosis group (AIS).

Rate of change of COM-COP inclination angle (RCIA,°/s)				
	AIS-V	P	AIS-A	P
Sagittal Plane				
CTO	-0.13	0.62	-0.47	0.07
CHS	-0.31	0.24	-0.02	0.94
TO	-0.47	0.07	-0.13	0.62
Average DLSi	-0.33	0.22	-0.50	0.05*
Average DLSt	-0.50	0.05*	-0.33	0.22
Average SLS	0.22	0.42	0.15	0.59
Average CSLS	0.15	0.59	0.22	0.42
Frontal Plane				
CTO	0.08	0.76	0.20	0.45
CHS	-0.12	0.64	-0.07	0.81
TO	-0.20	0.45	-0.08	0.76
Average DLSi	0.17	0.54	-0.12	0.66
Average DLSt	0.12	0.66	-0.17	0.54
Average SLS	-0.19	0.47	0.14	0.59
Average CSLS	-0.15	0.59	0.19	0.47

AIS-V: cycle of the convex-side limb; AIS-A: cycle of the concave-side limb; PV = AIS-V vs. Control; PA = AIS-A vs. Control; PS = AIS-A vs. AIS-V.

*: significant group effect; †: significant difference between sides (PS < 0.05); HS: heel-strike; TO: toe-off; CHS: contralateral heel-strike; CTO: contralateral toe-off; iDLS: initial double-limb support; tDLS: terminal double-limb support; SLS: single-limb support; CSLS: contralateral SLS.

## Discussion

The current study aimed to identify the effects of thoracic AIS on the whole-body balance control during level walking in terms of the motions of the COM relative to the COP, and its association with the severity of the spinal deformity. The results support the hypothesis that, when compared to healthy controls, the patients with AIS showed altered, compromised balance control. In the sagittal plane, compared to the healthy controls, the patients showed increased posterior RCIA over both the initial and terminal DLS for both limbs, i.e., the COP was moving faster towards the leading stance limb, indicating a more jerky transfer of the body weight over the gait cycle. In the frontal plane, the patients showed an increased IA magnitude over terminal DLS of the concave-side limb, suggesting greater sharing of the body weight on the convex-side limb. For between-side comparisons in AIS, differences occurred mainly in the frontal plane, with the concave-side showing more conservative balance control, as indicated by a reduced IA during SLS, and reduced sharing of the body weight with negative time-averaged IA (i.e., the average COP closer to the convex-side limb) during both initial and terminal DLS.

In the sagittal plane, the AIS group showed a jerkier control of the COM relative to the COP over SLS of the concave-side limb with an increased IA jerk index, suggesting increased difficulty in maintaining smooth balance control. During DLS, the AIS group increased the posterior RCIA for both limbs with the COP moving towards the leading stance limb at a faster and more varying speed than in the healthy controls, leading to a normal position control of the COM relative to the COP as indicated by the unaltered average IA over these phases. This could be related to the greater left rotation of the trunk and side-bending towards the concave side in the AIS group [14], which tended to induce a more posterior COM position towards the trailing limb, and thus an increased period with a more anterior position of the COP.

In the frontal plane, the increased bending of the trunk towards the limb on the concave side may minimize the separation of the COM and COP, leading to a COM-COP inclination angle smaller than that of the healthy controls. When the COM was moving away from the stance limb over the SLS, which will increase the neuromechanical demand for maintaining a smooth COM motion relative to the stance limb, the patients appeared to adopt a conservative strategy for a smooth balance control on the concave-side with a smaller IA over SLS and during contralateral toe-off and heel-strike. They were able to maintain balance during SLS of the convex-side limb similar to the Control.

During the terminal DLS phase, the COM was controlled within a relatively small range while the COP travelled from the ipsilateral limb to the contralateral limb during the body weight transfer. Therefore, the smooth motion of the COM relative to the COP in the frontal plane in terms of a smooth change of frontal RCIA, and normal or reduced IA jerk index is an indication of a well-controlled transfer of the body weight while maintaining dynamic balance. During the terminal DLS of the concave-side limb, the patients significantly increased the magnitude of the negative IA with unaltered RCIA. The patients with AIS were previously found to have a persistent trunk list towards the concave side [26], contributing to the changes of IA. This was also accompanied by a decreased duration of DLS or weight-release phase, during which the body weight was supported by the concave-side limb for shorter than by the other limb as indicated by the reduced period before the COP traveled to be right below the COM (i.e., zero IA). The altered body weight transfer during terminal DLS with increased IA magnitude but without an RCIA large enough (Table 2 and Table 4) for maintaining the dynamic stability of the COM [26] indicated an unstable balance control with an increased risk of loss of balance during weight release.

Generally, the patients with AIS adopted a conservative balance control strategy during walking with reduced performance in maintaining a smooth and stable transfer of the body weight and COM-COP control. Their COM-COP control was also affected by the severity of the spinal deformity, especially during SLS on the concave-side limb. During this period, the peak and average IA magnitudes were found to be moderately to strongly correlated with the Cobb angles, suggesting that the more severe the spinal deformity, the more conservative the balance control (i.e., reduced IA magnitudes) at the beginning and throughout SLS. The severity of the spinal deformity also affected the speed of the weight acceptance on the concave-side limb. The average posterior RCIA over the initial DLS (i.e., terminal DLS of the convex-side limb) was strongly correlated with the Cobb angles. The more severe the spinal deformity, the faster the COP was moving towards the leading concave-side stance limb, and thus the more jerky the COM-COP control. Therefore, this patient group, and especially those patients with a more severe spinal deformity, should be monitored for signs of an increased risk of loss of balance during weight transfer on the concave-side limb.

The current study was the first attempt to identify the effects of AIS on the control of the body’s COM motion relative to the COP in terms of IA and RCIA during level walking. The patient group was limited to female adolescents with Lenke 1 thoracic scoliosis without compensatory thoracolumbar curves in order to achieve better homogeneity of the patient group. Generalization of the current results to other patient groups should be made with caution. For the current patient group, the COM-COP control has been shown to be affected by the severity of the spinal deformity. Therefore, further studies should be extended to identify how the type of AIS, as well as the severity of the spinal deformity, might affect the COM-COP control during walking. On the other hand, muscle strength imbalance [16, 43, 44] is often found in patients with AIS and may limit the patient’s ability to control the trunk and COM-COP motion. A more complete knowledge of this information would be helpful for the management of patients with AIS. The IA and RCIA were proposed on the basis that the motion of the COM relative to the COP better describes the balance control during movement. This means the absolute positions and velocities of the COM and COP are not included, which may limit the comparison of the current results with those of previous studies using COM and COP data separately or using other kinetic and kinematic variables. Also, some strategies observed during standing balance control may not be revealed using only IA and RCIA [45]. Therefore, further studies may be needed to examine whether inclusion of absolute COM and COP data would help provide more insight into the balance control during gait in Lenke 1 patients.

## Conclusions

The patients with Lenke 1 thoracic scoliosis in the current study showed altered and jerkier COM-COP control during level walking when compared to healthy controls. During DLS, the patients increased the posterior RCIA in the sagittal plane with increased IA jerk index in the frontal plane for both the concave- and the convex-side limb, indicating their difficulty in maintaining a smooth transfer of the body weight. During SLS of the concave-side limb, the patients adopted a conservative COM-COP control strategy with decreased IA in the frontal plane but showed a jerky COM-COP control in the sagittal plane. The COM-COP control of the patients was associated with the severity of the spinal deformity. The more severe the spinal deformity, the faster the COP was moving towards the leading concave-side stance limb during DLS, and the more conservative the balance control throughout SLS of the concave-side limb, resulting in jerkier COM-COP control. Therefore, this patient group, and especially those with a more severe spinal deformity, should be monitored for signs of an increased risk of loss of balance during weight transfer on the concave-side limb.

## Supporting information

S1 Data (XLSX)
